# A targeted gene expression platform allows for rapid analysis of chemical-induced antioxidant mRNA expression in zebrafish larvae

**DOI:** 10.1371/journal.pone.0171025

**Published:** 2017-02-17

**Authors:** Margaret G. Mills, Evan P. Gallagher

**Affiliations:** Department of Environmental and Occupational Health Sciences, School of Public Health, University of Washington, Seattle, Washington, United States of America; University of Louisville School of Medicine, UNITED STATES

## Abstract

Chemical-induced oxidative stress and the biochemical pathways that protect against oxidative damage are of particular interest in the field of toxicology. To rapidly identify oxidative stress-responsive gene expression changes in zebrafish, we developed a targeted panel of antioxidant genes using the Affymetrix QuantiGene Plex (QGP) platform. The genes contained in our panel include eight putative Nrf2 (Nfe2l2a)-dependent antioxidant genes (*hmox1a*, *gstp1*, *gclc*, *nqo1*, *prdx1*, *gpx1a*, *sod1*, *sod2*), a stress response gene (*hsp70*), an inducible DNA damage repair gene (*gadd45bb*), and three reference genes (*actb1*, *gapdh*, *hprt1*). We tested this platform on larval zebrafish exposed to *tert*-butyl hydroperoxide (tBHP) and cadmium (Cd), two model oxidative stressors with different modes of action, and compared our results with those obtained using the more common quantitative PCR (qPCR) method. Both methods showed that exposure to tBHP and Cd induced expression of *prdx1*, *gstp1*, and *hmox1a* (2- to 12-fold increase via QGP), indicative of an activated Nrf2 response in larval zebrafish. Both compounds also elicited a general stress response as reflected by elevation of *hsp70* and *gadd45bb*, with Cd being the more potent inducer. Transient changes were observed in *sod2* and *gpx1a* expression, whereas *nqo1*, an Nrf2-responsive gene in mammalian cells, was minimally affected by either tBHP or Cd chemical exposures. Developmental expression analysis of the target genes by QGP revealed marked upregulation of *sod2* between 0-96hpf, and to a lesser extent, of *sod1* and *gstp1*. Once optimized, QGP analysis of these experiments was accomplished more rapidly, using far less tissue, and at lower total costs than qPCR analysis. In summary, the QGP platform as applied to higher-throughput zebrafish studies provides a reasonable cost-effective alternative to qPCR or more comprehensive transcriptomics approaches to rapidly assess the potential for chemicals to elicit oxidative stress as a mechanism of chemical toxicity.

## Introduction

Oxidative stress (OS) contributes to a wide range of human diseases, such as neurodegeneration [1–4], cancer [5–7], cardiac conditions such as hypertension and atherosclerosis [8–11], and cell death associated with ischemia/reperfusion injuries [12,13]. It is also a common mechanism leading to cellular damage caused by chemical exposure in humans [14–16] and wildlife species [17–21].

Response to cellular OS is mediated largely by the action of antioxidant proteins as well as low molecular weight thiols such as glutathione (GSH). Nrf2 (NF-E2 p45-related Factor 2), a CNC-bZIP (cap ‘n’ collar, basic-leucine zipper) family protein, is the primary transcription factor involved in mediating the induction of antioxidant proteins. Under homeostatic conditions, Nrf2 protein is bound in the cytoplasm by Keap1 (Kelch-like ECH associated protein 1) [22,23], where it is ubiquitinated and rapidly degraded [23–25]. During cellular oxidative stress, Nrf2 dissociates from Keap1 and translocates to the nucleus to associate with Maf proteins. This Nrf2-Maf heterodimer then binds to antioxidant response elements (AREs, also sometimes referred to as electrophile response elements, or EpREs) [26–28] to facilitate transcription of antioxidant genes. Prototypical Nrf2-responsive genes in mammalian cells include NAD(P)H-quinone oxidoreductase (*NQO1*), alpha class glutathione S-transferases (*GSTA1/2*), subunits of glutamate-cysteine ligase (*GCLC* and *GCLM*), as well as heme oxygenase (*HMOX*) [28–32]. While Nrf2 is not required for normal growth and development, animals deficient in Nrf2 activity are more susceptible to OS than animals with intact Nrf2 function [28,33].

Zebrafish have an intact Nrf2 pathway and a conserved set of genes that respond to oxidative stress [34], making them well-suited for investigations of chemical-mediated oxidative damage. The combination of their relatively large clutch sizes, ease of genetic manipulation, and rapid developmental timeline facilitates the ability to use zebrafish as a model to screen large numbers of compounds for toxicity [35–39]. However, to determine the ability of compounds to elicit an oxidative stress response in zebrafish, there is a need for methods that enable rapid analysis of biomarkers of oxidative stress with a higher throughput. Transcriptomic techniques such as RNAseq and microarrays are powerful methods to comprehensively identify molecular pathways perturbed by chemical exposures, but these approaches can be expensive and time-consuming. Assessing changes in expression of specific oxidative response genes by quantitative PCR (qPCR) can identify cellular oxidative stress and antioxidant responses, but this method is time-intensive and can be relatively cumbersome when analyzing large numbers of genes or samples, and is therefore poorly suited to higher-throughput analyses. In addition, results from qPCR analysis can be influenced by handling error, contamination, or variation in methodologies [40,41].

In the present study, we investigated the QuantiGene Plex (QGP) method, a multiplex branched DNA assay that eliminates the need for RNA isolation and reverse transcription, and allows for rapid quantification of many mRNAs from the same sample. In QGP assays, target-specific probes are bound to uniquely-labeled beads. This fluorescent bead-based technology allow for multiple transcripts to be processed together in the same well of a 96-well plate. The signal from each transcript is amplified using additional oligo probes that hybridize to the target and provide a scaffold for binding biotinylated label probes [42,43]. Quantification of the different mRNAs is done by correlating the unique bead fluorescence with the signal from label probes as measured by Luminex flow cytometer.

Our approach in comparing QGP with qPCR was to optimize a panel of zebrafish genes to assess the oxidative stress-related effects of toxicant exposures. These include known and putative Nrf2-responsive genes: those for which elimination of Nrf2 activity eliminates inducibility of expression (*hmox1a*, *gclc*, *gstp1*, *nqo1*) [28,33,44], or reduces but does not eliminate inducibility of expression (*gpx1a*, *prdx1*) [33,45], or eliminates inducibility by some compounds (*sod1*, *sod2*) [33,46–48]. Also included are a general stress-responsive gene (*hsp70*, responsive to Nrf2-dependent and -independent redox signaling and also to other forms of cellular stress) [49,50], and a DNA damage-inducible gene (*gadd45bb*) [31,51]. Three reference genes (*actb1*, *gapdh*, *hprt1*) were added to the panel to enable accurate standardization of expression [31,46,52,53]. Using this higher-throughput method, we analyzed the involvement of the Nrf2 pathway in response to oxidative stress generated by exposure to two model oxidative stressors, *tert*-butyl hydroperoxide (tBHP) and cadmium (Cd). These compounds can activate Nrf2 (e.g., [21,33]) but operate through different mechanisms. Specifically, tBHP is an organic hydroperoxide, which is hypothesized to directly damage cells by lipid peroxidation [33,54]. Cadmium is an electrophile that generates ROS and modifies Keap1, thus activating Nrf2 (reviewed in [55,56]). Since the multiplex platform of QGP has a higher-throughput capability than qPCR, we further tested QGP using a large sample size to examine gene expression during zebrafish larval development, and identified antioxidant genes that vary in either baseline expression or tBHP inducibility during this developmental period. Comparison of QGP to qPCR indicated that the two methods agree favorably on changes in gene expression after chemical exposures, but that QGP requires significantly less time and starting material to carry out the same analysis than qPCR, despite comparable reagent costs.

## Methods

### Fish care

All animal procedures were approved by the Institutional Animal Care and Use Committee of the University of Washington. Adult zebrafish of the outbred Ekkwill (EKW) strain were housed on a recirculating system, maintained at 27 +/- 1°C at a 14h:10h light:dark cycle. Fish received 2% of their body weight in flake food per day, and were supplemented with live *Artemia* at least once daily. Fish were allowed to spawn naturally at the start of the daily light cycle, and fertilized embryos were collected within a few hours.

### Chemical exposures

Zebrafish embryos and larvae were exposed to intended concentrations of 25 μM cadmium (as CdCl_2_, Mallinckrodt Baker, Phillipsburg, NJ) or 800 μM *tert*-butyl hydroperoxide (tBHP; VWR, Radnor, PA). The Cd concentration was based on an earlier study in our lab that showed a strong Nrf2 response in zebrafish larvae [21]. Similarly, the tBHP concentration employed was shown by colleagues to elicit a strong antioxidant response in zebrafish [57]. The test chemicals were diluted to these final concentrations in E3 embryo medium (0.1 mM NaCl, 3.4 μM KCl, 6.6 μM CaCl_2_, 6.6 μM MgSO_4_, pH 7.3). Exposures were carried out in 20 ml glass scintillation vials (Kimble-Chase, Rockwood, TN) each containing 10 ml of solution, with 8–10 fish per vial. For the acute exposures, fertilized embryos/larvae were grown in E3 embryo medium at 28°C until they reached 96 post fertilization (hpf). At 96 hpf, larvae were lightly anesthetized with MS-222 (Western Chemical, Ferndale WA), then added to solution vials. Larvae were maintained in solution vials at 28°C for 3 hours, then were collected as detailed below. For subacute time-course exposures, groups of 10 fertilized embryos were added to tBHP (800 μM) and control (0 μM) solution vials (20 vials each) at approximately 4 hours hpf and were maintained at 28°C. At 24 hpf, 5 vials each of tBHP and control exposures were collected as detailed below; the remaining replicates were given a 90% water change with pre-warmed solution. This process was repeated at subsequent 24 h intervals until all replicates had been collected at 96 hpf.

### RNA extraction and quantitative real-time PCR

For analysis of gene expression using qPCR, following chemical exposures, euthanized larvae were transferred to 2ml centrifuge tubes containing 700 μl Qiazol (Qiagen, Valencia, CA) and a 5mm steel bead (Qiagen), and the samples were homogenized in a TissueLyzer shaker (Qiagen) for 2 minutes at 40 Hz. RNA was extracted from homogenized fish and reverse transcribed into cDNA using iScript RT Supermix (BioRad, Hercules, CA) according to manufacturers’ protocols, using 1 μg RNA starting material per cDNA synthesis reaction. qPCR reactions were analyzed on a 7900HT Real-Time PCR System (Applied Biosystems, Foster City, CA). Cycling conditions were as follows: 95°C for 10’, followed by 40 cycles of denaturing (95°C) for 15” and annealing for 30”. Primer sequences and annealing temperatures are listed in Table 1. Each 20 μl reaction included 3 μM of each primer, 50 ng cDNA (RNA equivalent), and 1x SsoAdvanced SYBR Green Supermix (BioRad). Efficiency was calculated for each primer pair based on a 6-point standard curve of purified PCR product for that primer pair, amplified from a pool of cDNAs using GemTaq (MGQuest, Lynnwood, WA) and purified using the GeneJET Gel Extraction and DNA Cleanup Micro kit (Thermo Fisher). Standard curve samples were generated by 10-fold dilutions from 10 pg/μl to 0.0001 pg/μl. No-template and no-reverse-transcriptase samples were included as negative controls. Each sample was run in triplicate. The relative expression of each gene in each condition was calculated using the efficiency-based method [58]. The efficiency was calculated from the average CT (cycle threshold) readings for each point of the standard curve using the formula E = 10^(1/slope)^. Expression of genes of interest were normalized to the average of two stably-expressed reference genes (*actb1* and *gapdh*), calculated using the equation (1/E^CTgene^)/(1/E^CTreference^) or E^CTreference^ / E^CTgene^. To visualize the stability of each reference gene tested, 1/E^CT^ for each gene and for the geometric mean of the three were calculated and are presented in S1B Fig.

**Table 1:** Comparison of QGP and qPCR measurement of tBHP- and Cd-induced changes in gene expression in larval zebrafish.

Pathway	Gene	-Fold Induction: tBHP	-Fold Induction: Cd
Oxidative stress (known or putative Nrf2-responsive)	*hmox1a*	2.8 ± 0.7 **	11.5 ± 1.1 ****
		*2*.*2 ± 0*.*2* ***	*9*.*1 ± 0*.*7* ****
	*gclc*	2.5 ± 0.2 ****	3.5 ± 0.3 ****
		*2*.*7 ± 0*.*2* ****	*3*.*8 ± 0*.*3* ****
	*gstp1*	4.4 ± 0.3 ****	2.4 ± 0.1****
		*6*.*2 ± 1*.*6* ****	*3*.*8 ± 0*.*2* ****
	*nqo1*	1.3 ± 0.1 ***	1.2 ± 0.1 **
		*1*.*6 ± 0*.*5* *	*1*.*8 ± 0*.*2* ***
	*prdx1*	11.5 ± 0.7 ****	6.7 ± 0.4****
		*15 ± 2*.*0* ****	*8*.*1 ± 0*.*7* ****
	*gpx1a*	1.9 ± 0.3 ****	1.5 ± 0.1**
		*1*.*7 ± 0*.*2* ****	*1*.*4 ± 0*.*1* **
	*sod1*	0.96 ± 0.06	0.95 ± 0.05
		*1*.*0 ± 0*.*1*	*0*.*96 ± 0*.*09*
	*sod2*	0.91 ± 0.04 **	0.90 ± 0.03 **
		*1*.*07 ± 0*.*06*	*0*.*95 ± 0*.*03*
General stress	*hsp70*	8.2 ± 0.7 ***	33 ± 3 ****
		*5*.*6 ± 0*.*8* ***	*22 ± 2* ****
DNA damage-inducible	*gadd45bb*	1.4 ± 0.2 *	2.3 ± 0.2 ****
		*1*.*5 ± 0*.*4*	*2*.*4 ± 0*.*2* ****

Exposure to tBHP (800μM) or Cd (25 μM) for 3h at 4dpf induces expression changes of different magnitudes in response genes. For each gene, the -fold change in expression from control as induced by tBHP or Cd exposure is listed, as is the significance of the difference in expression change between the two chemicals. QGP results are given above in plain text; qPCR results are given below *in italics* and shaded grey. All data represent the mean ± SD of n = 5 biological replicates, 10 larvae per replicate, as measured by QGP.

Statistical significance is indicated as follows

* p < 0.05

** p < 0.01

*** p < 0.001

**** p < 0.0001. One-way ANOVA followed by Bonferroni’s multiple comparisons test.

### QuantiGene Plex

For analysis of gene expression using the QGP platform, larval fish were homogenized in 400 μl homogenizing solution including 4 μl Proteinase K (Affymetrix, Santa Clara, CA) as described for qPCR samples. Tubes of homogenized tissue were incubated at 65° C for 5 minutes, centrifuged at 14k rpm for 5 minutes, then incubated at 65° C for another 30 minutes with occasional vortexing. The samples were then centrifuged at 14k rpm for 10 minutes to pellet cellular debris. Supernatant samples were diluted in homogenizing solution: 1:4 for acute exposure and time course experiment, and further 1:1 serial dilutions for analysis of the linear range of the QGP assay. Diluted samples were stored at -80° C until used in the QGP assay, then heated to 37° C before use. The QGP assay was then carried out according to manufacturer directions using 40 μl of diluted homogenate or 500 ng purified RNA per well, with two technical replicates per biological replicate. Probes against genes listed in Table 1 were designed by Affymetrix. The incubations were carried out in a VorTemp shaking incubator (LabNet, Edison, NJ), and washes were accomplished with the aid of a magnetic plate holder (Invitrogen, Carlsbad, CA). Assay plates were then stored at 4° C overnight, brought to room temperature and analyzed on a MagPix reader (Luminex, Austin, TX). Median fluorescent intensity (MFI) readings were recorded for all samples. A baseline of twice the limit of detection (as recommended by the manufacturer) was used as the minimum signal allowed for the gene analyzed. Expression of genes of interest were normalized by dividing by the geometric mean of three reference genes (*actb1*, *gapdh*, and *hprt1*) except when comparing QGP results to qPCR results, in which case expression was normalized by dividing by the average of two reference genes (*actb1* and *gapdh*).

### Statistical analysis

All statistical analyses were conducted using GraphPad Prism version 6. Gene expression data are expressed as the mean ± SD of n = 5 pools of 10 larvae for each treatment and condition except where noted. Relative–fold induction was calculated by dividing the average normalized values of the treated samples by the average normalized value of the control samples. The effects of chemical exposure on gene expression were assessed in using one-way ANOVA followed by a Bonferonni correction for multiple comparisons. To compare agreement between QGP and qPCR methods, each average -fold change in gene expression was used as a single data point for correlational analysis.

## Results

### Gene expression results are comparable for both QGP and qPCR

**Fig 1:**
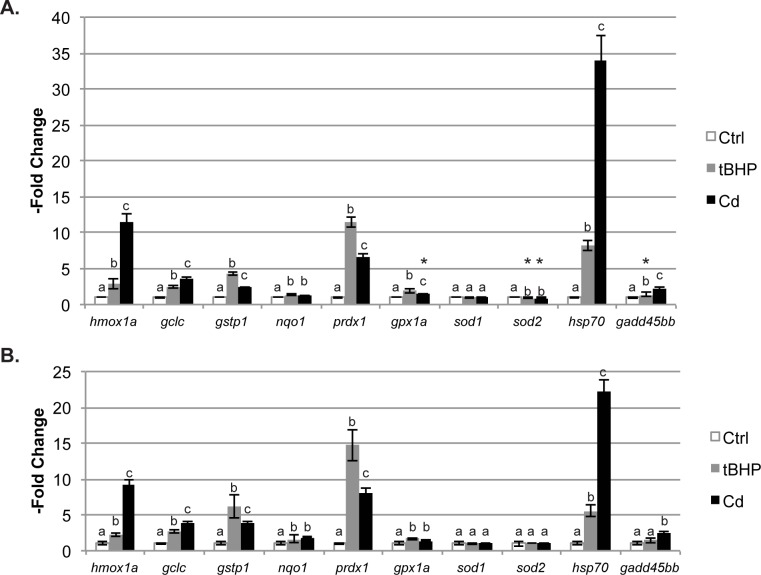
Acute exposure to tBHP or Cd induces differential changes in gene expression in larval zebrafish, as measured by QGP and qPCR. (A) As measured by QGP, the genes for which expression is affected by 3h exposure to tBHP (800 μM) or Cd (25 μM) at 4 dpf are the same, but the magnitude of the induction varies significantly. (B) Similar changes in gene expression are measured by qPCR as compared to QGP. All data represent the mean ± SD of n = 5 biological replicates, 10 larvae per replicate. For each gene, letters above the bars indicate results significantly different at p < 0.05. * indicates treatments that were significantly different in the QGP assay, but not by qPCR.

**Fig 2:**
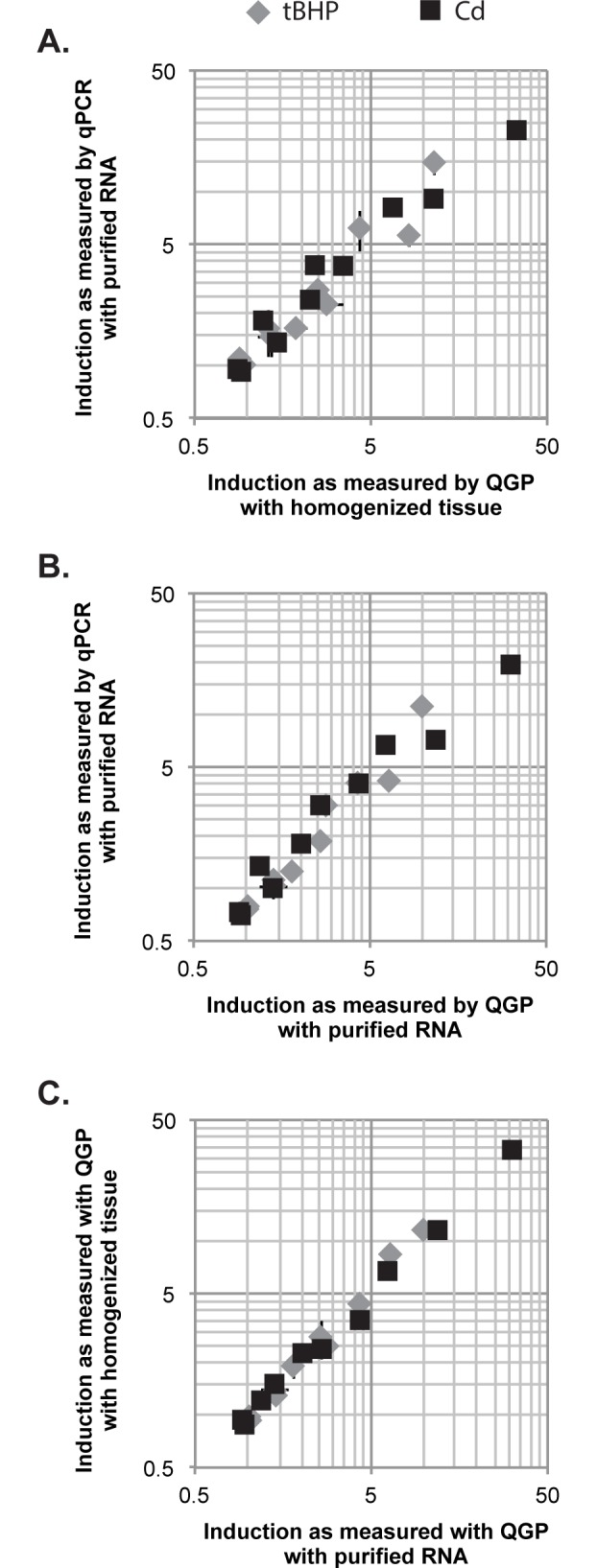
The differences in–fold induction measured by QGP and qPCR are due to quantification method rather than tissue processing method. (A) Gene expression in separate pools of larvae is substantially similar between QGP and qPCR methods, as measured by linear regression (y = 0.68x + 1.20, R^2^ = 0.902). Each point represents a particular gene as affected by exposure to either tBHP or Cd, showing the induction of expression as measured by QGP (X-axis position) and qPCR (Y-axis position). All data represent the mean ± SD of n = 5 biological replicates, 10 larvae per replicate. (B) Gene induction in purified RNA of the same two biological replicates as measured by QGP (X-axis position) and qPCR (Y-axis position) follows the same pattern as that seen in (A), with similar agreement between by the two methods as measured by linear regression (y = 0.64x + 0.74, R^2^ = 0.926). All data represent the mean ± SD of n = 2 biological replicates, 10 larvae per replicate. (C) Gene induction in purified RNA (X-axis position) or homogenized tissue (Y-axis position), both measured by QGP, are more similar to each other than the comparisons in either (A) or (B) as measured by linear regression (y = 1.08x – 0.13, R^2^ = 0.995). Data represent the mean ± SD of n = 2 biological replicates for RNA and n = 5 biological replicates for homogenized tissue, 10 larvae per replicate for both. Many error bars are too small to be visible beyond the symbols for each data point.

**Fig 3:**
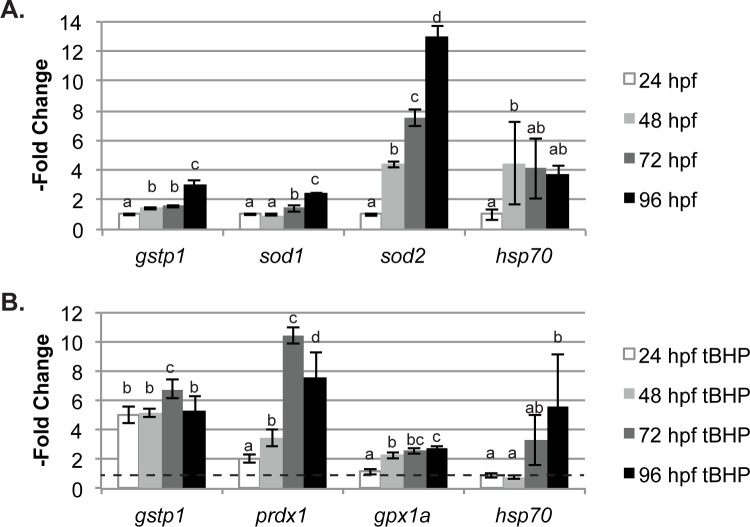
QGP analyses show baseline and tBHP-induced changes in expression of oxidative response genes through the first four days of zebrafish development. (A) Changes in expression of the four most strongly-affected genes in untreated fish. Expression of each gene is graphed relative to measured levels at 24 hpf for that gene. For each gene, letters above the bars indicate results significantly different from each other at p < 0.05 (B) Changes in expression of the four genes most strongly induced by subacute exposure to tBHP starting at 4 hpf. Expression of each gene is shown as–fold change relative to the untreated control from the same time point (dotted line). For each gene, letters above the bars indicate results significantly different from each other and from the untreated expression (group a, also indicated by dotted line) at p < 0.05. All data represent the mean ± SD of n = 5 biological replicates, 10 larvae per replicate.

To compare gene expression results from the QGP and qPCR platforms, a panel of 10 genes of interest and 3 reference genes were assessed in larval zebrafish at 4 days post fertilization (dpf) with and without exposure to tBHP and Cd. The three reference genes (*actb1*, *gapdh*, and *hprt1*) were stably expressed across exposure conditions as measured by QGP and at levels similar to those of our genes of interest (S1 Fig). However, *hprt1* was not stably expressed across exposure conditions as measured by qPCR, as shown in S1B Fig. Thus, in order to directly compare QGP and qPCR results, all gene expression data were normalized to the average of *actb1* and *gapdh* expression and are presented in Figs 1 and 2. For further analyses of QGP data alone (Fig 3), we used the geometric mean of all three reference genes to normalize expression of all genes of interest in our QGP assay.

Overwhelmingly, the observed patterns of baseline gene expression and -fold induction were highly comparable between the two platforms (Fig 1, Table 1). For most genes, both QGP and qPCR results agree on whether or not expression changes significantly with tBHP and/or Cd exposure, and on the direction of change (induction vs. repression). The magnitude of -fold induction, however, differed for a few genes. Specifically, *hsp70* induction for both tBHP and Cd was higher as measured by QGP (33-fold induction by QGP; Fig 1A, Table 1) than as measured by qPCR (22-fold induction; Fig 1B, Table 1), whereas the reverse was true for *prdx1* (11-fold induction for QGP vs. 15-fold induction for qPCR, Table 1). No difference in results was observed using either method of normalization in our analysis of the QGP data.

When QGP and qPCR yielded conflicting results on the significance of changes in gene expression, the–fold changes in question were minor. For example, while expression of *sod2* was unaffected by either tBHP or Cd exposure as measured by qPCR, analysis by QGP shows a repression that is significant, but only by 0.9-fold, p<0.001, for both chemicals (Table 1). For only one gene (*gpx1a*) did the two methods disagree on whether Cd or tBHP induces a larger -fold change in expression. Specifically, QGP revealed that tBHP induces a greater–fold change than observed for Cd (1.9-fold for tBHP vs. 1.5-fold for Cd, p<0.01), while qPCR found that the -fold changes do not significantly differ (1.7-fold for tBHP vs. 1.4-fold for Cd, p = 0.08).

Overall, we found that the two methods return similar, albeit not identical, results on the effects of tBHP and Cd exposure on gene expression in larval zebrafish (Fig 2A). We next wanted to assess where the difference is occurring between the two methods. Since the QGP assay can eliminate the need for RNA isolation, we evaluated whether the difference in results stemmed from tissue processing or from quantification. To do so, we used purified RNA from the same samples we had used in qPCR analysis as a template in the QGP assay. We then compared the measured -fold induction from these samples (QGP run on purified RNA) to the two other data sets: qPCR analysis of purified RNA (Fig 2B) and unpurified samples run on QGP (Fig 2C). There was a higher correlation between the two QGP results when using different samples (e.g., RNA vs. homogenized tissues; Fig 2C) than between the QGP and qPCR results from the same RNA samples (Fig 2B). Therefore, the differences observed between QGP and qPCR appeared to be due to differences in the quantification methods, as opposed to the tissue processing steps that precede mRNA quantification.

### Differential effects of acute tBHP and Cd exposure on gene expression in larval zebrafish

Once we confirmed that the QGP platform yielded comparable results to qPCR, we further analyzed the results from our QGP assay for the analysis of the effects of the two model oxidative stressors on antioxidant gene expression (Fig 1A). Both chemicals induced significant changes in expression of the same nine genes of interest (e.g. all but *sod1)* in our targeted QGP panel. Exposure to both tBHP and Cd resulted in a significant but minor decrease in *sod2* expression (10%) as measured by QGP, whereas all other gene expression modulations were consistent with gene inductions. Acute exposure to each compound induced different patterns of specific gene expression, with the extent of gene induction differing between tBHP and Cd for seven of the affected genes (*hmox1a*, *gclc*, *gstp1*, *prdx1*, *gpx1a*, *hsp70*, and *gadd45bb*; Table 1). Some of these genes were more strongly induced by tBHP (*gstp1* and *prdx1* in particular), while others responded more strongly to Cd (*hmox1a* and *hsp70* in particular).

### Changes in gene expression and inducibility by tBHP through embryonic and early larval development

We leveraged the multiplex capabilities of QGP to carry out a time-course exposure analysis that would be much more time-consuming to process with qPCR. Our objective was to determine how responses to oxidative stress differ over time as zebrafish develop from early embryos to larvae. To do so, we tracked the expression of our QGP panel of antioxidant genes through the first four days of zebrafish development both in untreated fish (Fig 3A and S2A Fig) and in fish exposed to tBHP (Fig 3B and S2B Fig). As observed in Fig 3, the baseline expression of several genes changed through this developmental period. For example, *gstp1* and *sod1* expression increased from 24 hpf to 96 hpf (Fig 3B). Expression of *gstp1* increased 3-fold (± 0.3; p<0.0001) while *sod1* increased 2.46-fold (± 0.04; p<0.0001) and *hsp70* increased to 3.7-fold (± 0.7, p<0.0001). The most dramatic observed change was in *sod2* expression, which increased steadily through the first 96 hours, reaching a 13-fold (± 0.6; p<0.0001) increase over 24 hpf level (Fig 3A).

The inducibility of genes by subacute tBHP exposure varied tremendously (Fig 3B and S2 Fig). Exposure to tBHP induced expression of *gstp1* between 5- and almost 7-fold over their time-matched controls at every time point. Several genes, most notably *prdx1*, showed both significant induction and also significant changes in inducibility by tBHP during the time course experiment, with expression of *prdx1* in tBHP-exposed fish increasing from not significantly different than time-matched control at 24 hpf to over 10-fold that of control at 72 hpf (Fig 3B). In contrast, *sod2* showed no induction by tBHP at any point analyzed (S2B Fig).

### Comparative analysis of costs, time, and tissue requirements for QGP and qPCR

Because the protocols for QGP and qPCR differed so dramatically, direct comparison of the costs associated with the two methods was difficult. Therefore, we undertook a comprehensive analysis of the cost of all reagents used for each method from euthanizing whole larvae to acquiring gene expression data as described in our Methods, the amount of time required for these steps, and the amount of larval zebrafish tissue required to obtain that data. The results of this analysis are presented in Table 2; detailed lists of reagents (including product numbers and prices) used to calculate these numbers are provided in S1 Table. For this analysis, we calculated costs for experiments involving analysis of two different numbers of total genes analyzed (e.g. 5 genes, a reasonable number of genes in studies of oxidative stress responses; or 13 genes, the number of genes that we included in our panel). Also analyzed were 3 different numbers of biological samples (e.g. 15 samples, the number of samples in our acute exposure experiment; 40 samples, the number of samples in our time-course experiment; or 200 samples, representing a hypothetical larger-scale comparison of treatment effects). Reagent costs for qPCR vary tremendously depending on the specific manufacturers and kits used (see S1 Table for details), but we observed that the reagent cost for QGP assays falls within or below those qPCR costs for all numbers of samples, and also when analyzing the expression of 13 genes. By contrast, when a project calls for analyzing only a few genes of interest for a particular project, QGP is a less favorable choice. This is because reagent costs are as high, or higher, than those for qPCR for equivalent numbers of samples. However, it is important to note that QGP still offers an advantage in the amount of time required (Table 2) as well as greater simplicity.

**Table 2:** Comparison of reagent costs and time required to analyze gene expression using QGP or qPCR with SYBR Green chemistry.

		Reagent Cost^a^		Labor^b^		Total time^c^	
		QGP	qPCR^d^	QGP	qPCR	QGP	qPCR
5 genes	15 samples	$807	$741–831	2.9	6.5	6.3	15.1
	40 samples	$1,877	$1,473–1,797	3.0	8.6	6.5	22.3
	200 samples	$8,240	$5,989–7,308	3.9	23.0	17.7	70.4
13 genes	15 samples	$1,261	$1,528–1,759	2.9	10.1	6.3	26.3
	40 samples	$2,998	$2,941–3,568	3.0	15.8	6.5	45.7
	200 samples	$13,969	$11,806–14,356	3.9	51.7	17.7	169

See S1 Table for details of the calculations used to generate these numbers.

^a^ All amounts listed are calculated using list prices. Of note is that most companies offer a 15% discount on these prices to academic institutions. Additional costs involving equipment, and variables such as owning or renting time on qPCR thermal cyclers, are not included, but will affect the total costs for a given lab.

^b^ Calculated as the number of hours of hands-on time required to complete the assays. Does not include incubations longer than 10 minutes in length.

^c^ Calculated as the total number of hours required to complete the assays, including incubations under 8 hours in length.

^d^ Reagents from two different suppliers were used to calculate a range of prices.

We calculated the amount of time required to perform all of the steps necessary for each method, including that required for “sample prep” (homogenizing larvae, and purifying RNA and generating cDNA for qPCR), “standard prep” (generating standard curves used in qPCR), and quantification of gene expression (the qPCR or QGP assays themselves). For this, we tabulated both the total time and the “hands-on time” (listed as “Labor” on Table 2), which excludes any incubation step over 10 minutes in length, when the researcher could be working on other projects. The amount of time involved in running the different assays varied extensively based on the number of genes and biological samples tested. In all cases, however, QGP required less hands-on time and total analysis time when compared to qPCR (Table 2). Analysis of all genes in the QGP panel can be completed in 1.5 days for up to 92 biological samples, including three one-hour breaks in the longer day of processing (an incubation of 18–22 hours separates the initial half-day from the subsequent full day, and this period is excluded from the total time calculation.) The difference in total time for each assay includes the amount of time required to run samples on quantification machines. Each QGP plate, which can hold up to 46 biological samples and analyze the expression of ≤ 80 genes, can be run on a Luminex flow cytometer in an hour. In contrast, each qPCR reaction plate can hold up to 32 biological samples and requires an hour per single gene analyzed.

A noteworthy finding from our comparison of the two methods was the relative simplicity of the QGP assay compared to qPCR. For example, QGP does not require the processing steps needed for purifying RNA and generating the cDNA templates required for qPCR, thus eliminating many of the potential sources of variation and contamination in the qPCR protocol. Because QGP is a multiplex assay, expression of all genes from a given biological replicate are measured in the same well. As a result, analysis of relative gene expression is not subject to error caused by the variable loading of template or quantification reagents into the wells for a particular gene, as can occur with qPCR.

**Fig 4:**
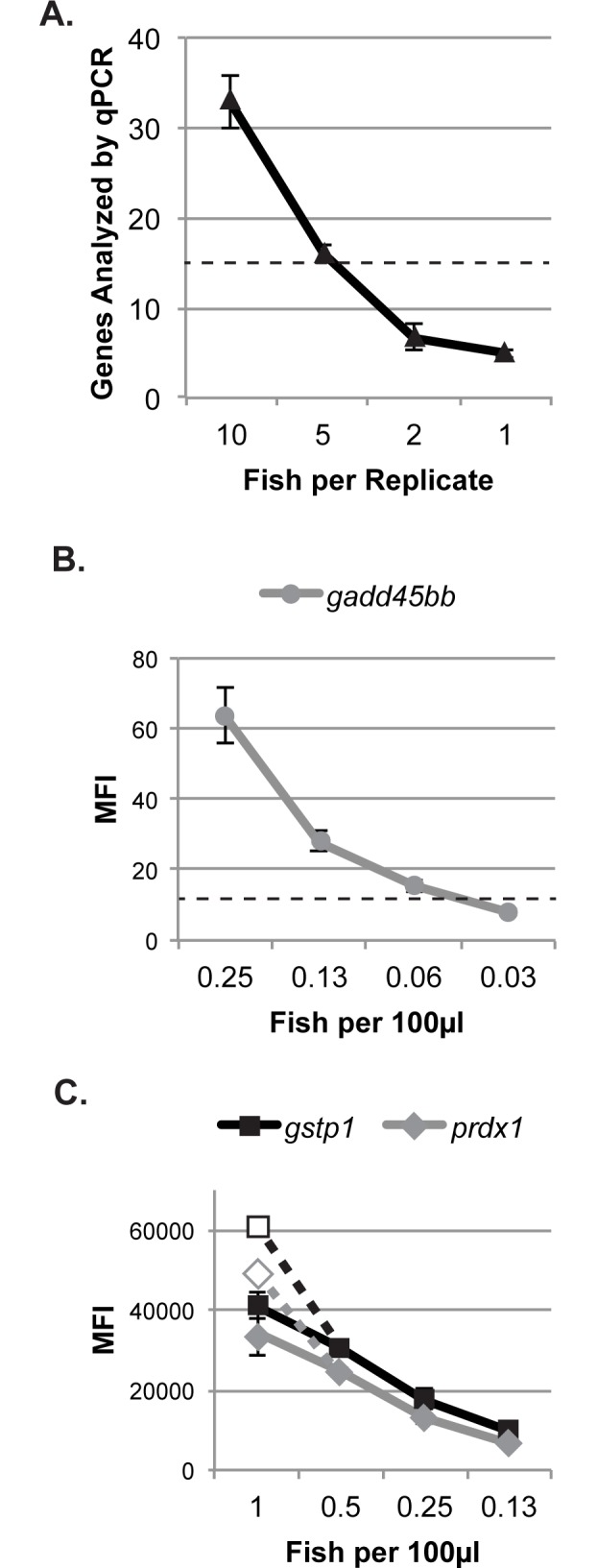
Analysis of the amount of 4 dpf zebrafish tissue necessary for expression analysis using qPCR or QGP. (A) RNA was isolated from pools of varying numbers of larvae, and the maximum number of genes that could be analyzed via qPCR was calculated from the amount of purified RNA obtained. RNA concentration falls below the minimum needed to proceed with cDNA synthesis (dotted line) with fewer than 4 fish per biological replicate. (B-C) Serial dilutions of homogenized tissue in homogenization solution were analyzed with QGP. (B) For control fish, mean fluorescent intensity (MFI) recorded for of *gadd45bb* falls below 2x LOD (dotted line) at a dilution of 0.03 fish per 100 μl, the volume of homogenized tissue necessary to analyze a single QGP plate. (C) For tBHP-treated fish, MFI of *gstp1* and *prdx1* is saturated at 1 fish per 100 μl but not at 0.5 fish per 100 μl. (Dashed lines and outlined symbols show expected readings for 1 fish per 100 μl based on the readings from 0.5 fish per 100 μl.) All data represent the mean ± SD of n = 3 biological replicates.

QGP analysis can also be carried out on far less tissue than qPCR. Measuring gene expression by qPCR as described here requires 4 x 50ng, or 200 ng RNA-equivalent, per gene analyzed (using 50 ng of RNA-equivalent cDNA in each reaction and 3 technical replicates per biological replicate, plus about 50ng from each biological replicate to generate standard curves and a No RT negative control). We extracted RNA from replicate pools of varying numbers of 4 dpf larvae, measured the concentration of the purified RNA, and calculated the maximum number of genes that could be analyzed by qPCR using the resulting cDNA (Fig 4A). Carrying out cDNA synthesis as described here (using iScript RT supermix, using 1 ug RNA per reaction) requires a minimum concentration of 1 μg/16 μl, or 62.5 ng/μl RNA. Based on these data, we conclude that a minimum of 4 larvae per biological replicate are necessary to analyze the 13 genes in our panel, and this would leave little to no RNA for future analyses. In contrast, analysis of all genes in our QGP assay was carried out using the equivalent of a fraction of one larval zebrafish per biological replicate. Expression of the most low-expressed gene in our panel, *gadd45bb*, was measurable even when the homogenized tissue was diluted to the equivalent of 0.06 fish per 100 μl homogenization buffer (a volume large enough to run the sample on one QGP plate) (Fig 4B), therefore a single 4 dpf larva contains enough tissue to analyze our panel of 13 genes in 16 QGP assays. At the other end of the detection limit, expression of the two most highly-expressed (and most highly-induced) genes in our panel, *gstp1* and *prdx1*, was saturated at a concentration of 1 larva per 100 μl homogenization buffer (Fig 4C). In summary, gene expression from individual zebrafish larvae can easily be analyzed in this panel while still leaving additional material for analysis with future gene expression panels. This enables the use of fewer animals per assay than with qPCR analysis, while allowing for analysis of individual variation in gene expression.

## Discussion

In the current study we show that analysis of antioxidant gene expression by QuantiGene Plex (QGP) is comparable to that of qPCR. QGP probes have been validated for use in other species [42,43,59], but ours is the first published use of the assay for quantifying expression of zebrafish genes. Despite comparable reagent costs, the QGP assay required less (and often vastly less) time and tissue to complete, and allowed us to avoid the lengthy and contamination-prone steps involved in qPCR. We found that while the two methods did not agree on the precise–fold changes in expression induced by exposure of larval zebrafish to tBHP or Cd, they did agree on the particular genes whose expressions were significantly modulated by two prototypical oxidative stressors, and the extent of -fold change in gene expression. The relative effects of the two chemicals, whether tBHP or Cd induced a greater change in expression of a particular gene, was also consistent between the two platforms, with a few exceptions for which the biological significance is probably low, as these cases all involve either increases in gene expression of less than 2-fold (in the cases of *nqo1* and *gadd45bb*) or a decrease in expression to 0.9x (in the case of *sod2*). We also conclude that the optional step of purifying RNA from zebrafish tissue before use in the QGP assay is not necessary and does not provide any advantage in comparing QGP results to qPCR results.

There are several possible explanations for the difference in absolute–fold change observed by these two quantification methods. For some genes, the qPCR primers we used amplified a region of the gene entirely overlapping that covered by QGP probes; for others, the gene regions did not overlap at all (see S1 File for locations of all probes). Differences in the regions to which probes anneal may explain some of the disagreement in -fold change as measured by QGP versus qPCR. In those cases, it is possible that the probes may be measuring different splice forms of the same gene, or differently measuring closely-related paralogous genes. The zebrafish genome contains many fewer transcripts that undergo alternate splicing than the genomes of most other vertebrates [60], but it has a correspondingly higher proportion of duplicate gene pairs [61,62], which may differentially affect the ability to measure expression from one gene versus two or more closely related genes with the two methods. For example, our QGP probes were designed against *gstp1*, but these may also bind to and measure the closely-related *gstp2*. *gstp1* and *gstp2* share 90.4% sequence identity in zebrafish [63], and are thus extremely difficult to distinguish using oligo-based methods [31,46]. QGP probes bind to 400 nucleotides or more of mRNA for each gene, making it more difficult to target individual members of some paralog pairs with QGP than with qPCR primers. In contrast, one of our qPCR primers was designed to anneal in the 5’ UTR to allow specific amplification of *gstp1* alone [21,64]. However, for two of the genes that showed the greatest difference in–fold change measured by the two methods (*hmox1a* and *prdx1*), the qPCR primers used amplified a region mostly, or entirely, overlapping with that bound by the QGP probes. Because different probes were used in quantification of RNA by QGP and qPCR methods, those probes might reasonably be expected to differ in the efficiency with which they bind to mRNAs (e.g., [65,66]). These explanations for the observed differences in measured–fold changes in expression of individual genes could be tested by designing additional qPCR primers that overlap with the regions bound by QGP probes. By contrast, probes for *gclc* yielded nearly identical results for the two methods despite binding to entirely different regions of the transcript (Table 1, S2 Fig). However, as is the case for the use of qPCR to confirm changes in expression measured by microarray (e.g., [31,67]), the exact–fold change is of less interest than the identity of genes affected by exposure and the direction of change elicited. Accordingly, the QGP method is a reasonable cost- and time-effective option to ascertain whether a test chemical or drug induces an oxidative stress response in zebrafish larvae.

Analysis of our QGP data showed that acute exposure of larval zebrafish to Cd generated changes in gene expression, many of which were similar to those in previous reports of Cd exposures in zebrafish. For example, we previously reported that Cd exposure induced expression of *gstp1*, *gclc*, *hmox1a*, and *prdx1* in 4 dpf zebrafish [21]. In addition, we provide quantitative confirmation of previous qualitative reports of Cd-elicited induction of hsp70 by Cd [68,69], and further novel evidence of induction of *gpx1a* and *gadd45bb*.

Although tBHP is a known generator of oxidative stress (e.g., [33,70–73]), the only published report on the effects of tBHP on expression antioxidant genes showed that exposure of zebrafish embryos from 24 to 48 hpf increased expression of *gclc*, *gstp*, *gpx1* (identified as *gpx1a* by us), while neither *sod1* nor *sod2* were significantly affected by tBHP [46]. We confirmed that acute exposure to tBHP at 4 dpf had a similar modulating effect on expression of *gclc*, *gstp1*, and *gpx1a*, and further identified *prdx1* and *hsp70* as genes that show a greater responsiveness to this exposure. Notable was that the effects of tBHP on zebrafish gene expression varied depending on the developmental timing and length of exposure, which has been reported for *tert*-butylhydroquinone [31] certain polycyclic aromatic hydrocarbons [46], and fullerene [67]. While *hmox1a* expression increased after 3 h exposure to tBHP, no significant increase was measured after longer exposures (S2 Fig). These results are consistent with previous reports that expression of *hmox1a* is upregulated and then return to baseline quickly after exposure to oxidative stress [74].

It was interesting that two genes previously reported to be modulated by Cd or tBHP exposures in mammalian species (*sod2* and *nqo1*) were insensitive to modulation in embryonic/larval zebrafish in our studies. Our results suggest that there exist marked differences in transcriptional control for these genes between zebrafish and mammals. For example, expression of *sod2* was not substantially affected by either Cd or tBHP exposure (Fig 1, S2 Fig, in agreement with [46]), unlike previous reports in mammals [75,76]. Secondly, while *nqo1* expression is one of the most robust markers of Nrf2 activation in mammalian tissues [28,77,78], and is induced in human cells by Cd [79], it does not appear to be as widely inducible in zebrafish (Fig 1 and [31,46]). Rousseau et al. report that the zebrafish *nqo1* promoter lacks ARE sites, and also that *nqo1* expression in zebrafish is higher in fish with *nrf2*^*fh318*^ mutant alleles than in wild-type fish [80].

Of note in our developmental time-course analysis was that *gstp1* and *sod1* showed moderate increases in baseline expression during the first 96 hpf (which had been suggested by previous results [31]), and *hsp70* expression increased four-fold between 24 hpf and 48 hpf. Most noteworthy though, we found that baseline expression of *sod2* increased dramatically during the first four days of development. This change in baseline expression may indicate a substantial difference in the role of *sod* genes in embryonic and larval stages. In any case, it is important that these critical developmental timelines be accounted for in experiments on *sod* expression or function during zebrafish development. Identifying the transcriptional control of zebrafish *sod2*, in particular, would inform other research into the development of antioxidant function in larval zebrafish.

In conclusion, we assembled a panel of antioxidant genes and reference genes that allowed us to examine the oxidative stress effects of chemical exposure and development on their expression in zebrafish tissue. With these results, we were able to quickly identify chemicals that were potential activators of the Nrf2response, and to identify gene expression patterns in OS response. The differences in gene modulation by two model compounds analyzed provide insight into the different mechanisms by which these two model oxidative stressors cause cell injury, as well as activate Nrf2. By contrast, surveying a broad range of putative Nrf2-responsive genes in zebrafish exposed to multiple compounds and concentrations is more cumbersome task when approached using qPCR. Ultimately, transcriptomics approaches provide a more comprehensive analysis of chemical induced oxidative stress, but may not be either cost–or time–effective when applied to analysis of multiple chemicals. Furthermore, because QGP analysis can be completed using such small quantities of tissue, this panel can be used to examine individual variation in expression of oxidative stress response genes, such as in different inbred or outbred zebrafish strains. Collectively, these aforementioned possibilities make the QGP panel a powerful tool for characterizing oxidative stress responses in zebrafish.

## Supporting information

S1 Fig QGP and qPCR measure similar relative levels of expression from three reference genes in 4 dpf zebrafish larvae. Expression from 4 dpf zebrafish larvae exposed to either tBHP (800 μM) or Cd (25 μM) for 3h, as measured by **(A)** QGP and **(B)** qPCR. See Methods for details of the y-axis measure used for each method. As measured by both methods, expression of *actb1* and *gapdh* do not change significantly with exposure to either chemical. *hprt1* expression with Cd exposure changes only as measured by qPCR (p<0.05), not by QGP. The geometric means of the three reference genes do not change significantly as measured by either method. **(C)** Raw (un-normalized) expression for all genes as measured by QGP show that the reference genes are expressed at levels similar to the genes of interest in the panel. All data represent the mean ± SD of n = 5 biological replicates, 10 larvae per replicate.
(EPS)

S2 Fig QGP analysis of expression of oxidative response genes of control and tBHP-exposed zebrafish through the first four days of development. **(A)** Overview of expression of all 10 genes of interest in untreated control fish. Expression of each gene is graphed relative to measured levels at 24 hpf for that gene. **(B)** Overview of expression of all 10 genes of interest in zebrafish embryos exposed to 800 μM tBHP starting at 4 hpf. Expression of each gene is shown as–fold change relative to the untreated control from the same time point (dotted line). All data represent the mean ± SD of n = 5 biological replicates, 10 larvae per replicate.
(EPS)

S1 File Sequence and probe information for genes analyzed in this paper. For each gene in our oxidative stress response panel, we list: accession number for the transcript; qPCR primer sequences; fragment size generated by those primers; annealing temperature, efficiency, and source (if any) for those primers; and the complete transcript sequence for the gene, showing binding locations of all QGP and qPCR oligos. Genes are listed in alphabetical order.
(DOCX)

S1 Table Calculations used to generate reagent cost and time numbers listed in Table 1. For each set of conditions (number of samples and number of genes analyzed), a separate tab lists all the specific reagents and assumptions used to calculate the amount of time and money required for analyzing each hypothetical experiment by QGP or qPCR. All costs for kits and plates are prorated. For example, TissueLyzer processing of 15 samples requires 7.5% of a 200-bead pack, therefore we include 7.5% of the $181 cost for that pack.
(XLSX)
