# Supplementary material for: A targeted gene expression platform allows for rapid analysis of chemical-induced antioxidant mRNA expression in zebrafish larvae
Source: PLoS One. 2017 Feb 17;12(2):e0171025. doi: 10.1371/journal.pone.0171025 (PMC5315391; doi:10.1371/journal.pone.0171025)
Supplement: S1 File — For each gene in our oxidative stress response panel, we list: accession number for the transcript; qPCR primer sequences; fragment size generated by those primers; annealing temperature, efficiency, and source (if any) for those primers; and the complete transcript sequence for the gene, showing binding locations of all QGP and qPCR oligos. Genes are listed in alphabetical order. (DOCX) [file pone.0171025.s003.docx]

**S1 File.** **Sequence and probe information for genes analyzed in this paper.** For each gene in our oxidative stress response panel, we list: accession number for the transcript; qPCR primer sequences; fragment size generated by those primers; annealing temperature, efficiency, and source (if any) for those primers; and the complete transcript sequence for the gene, showing binding locations of all QGP and qPCR oligos. Genes are listed in alphabetical order.

QGP oligo locations are color coded:

CE is BLUE (oligos that connect the transcript to the bead), LE is RED (oligos that connect the transcript to the bDNA system to produce signal) and BL is green (oligos that fill space between CE and LE oligos)

qPCR oligo locations are underlined

***actb1***

>gi|18858334|ref|NM_131031| Danio rerio actin, beta 1 (actb1), mRNA.

F primer: ACATCCGTAAGGACCTG

R primer: GGTCGTTCGTTTGAATCTC

Fragment size: 319 bp

Annealing temperature: 50° C

Efficiency: 1.91

Source: [1]

ggcacgagagatcttcactccccttgttcacaataacctactaatacacagccatggatgaggaaatcgctgccctggtcgttgacaacggctccggtatgtgcaaagccggttttgctggagatgatgcccctcgtgctgttttcccctccattgttggacgacccagacatcagggagtgatggttggcatgggacagaaagactcctatgtgggagatgaggctcagagcaagagaggtatcctgaccctcaaataccccattgagcacggtattgtgaccaactgggatgacatggagaagatctggcatcacaccttctacaatgagctccgtgttgcccctgaggagcaccctgtcgtgctcactgaggctcccctgaatcccaaagccaacagagagaagatgacacagatcatgttcgagaccttcaacacccctgccatgtatgtggccatccaggctgtgctctctctgtacgcttctggtcgtactactggtattgtgatggactctggtgatggtgtgacccacaccgtgcccatctatgagggttacgctcttccccatgccatcctgcgtctggatctagctggtcgtgacctgacagactacctgatgaagatcctgaccgagcgtggctacagcttcaccaccacagccgaaagagaaattgtccgtgacatcaaggagaagctgtgctatgtggccctggacttcgagcaggagatgggaaccgctgcctcttcttcctccctggagaagagctatgagctgcctgacggtcaggtcatcaccatcggcaatgagcgtttccgttgccccgaggctctcttccagccttccttcctgggtatggaatcttgcggtatccacgagaccaccttcaactccatcatgaagtgcgacgtggacatccgtaaggacctgtatgccaacacagtgctgtctggaggtaccaccatgtaccctggcattgctgaccgtatgcagaaggaaatcacctctcttgctccttccaccatgaagatcaagatcattgctccccctgagcgcaaatactccgtctggatcggtggctccatcttggcctccctgtccaccttccagcagatgtggatcagcaagcaggagtacgatgagtctggcccatccatcgttcacaggaagtgcttctaaacagaactgttgccaccttaaatggcctagcaatgagattcaaacgaacgaccaacctaaactctcgaacagaacaagatgacatcagcatggcttctgctctgtatggcgcattgactcaggatgcggaaactggcaaagggaggtagttgtctaacaggggagagctttccccgagaggacaacaatgtacatttcttttagtcattccagaagcgtttaccactttgccctcctcacaatgggcgtccatgacctttttgttatagtgttttatgtaaattatgtactcgatacattgtttttctttttgtacttcagccttaaacttggcccagtttgttattgttgcaatgaggggaaagctttaccttttaaaaagtgaagatcttgcaggacttccctagggtatgtgaataagggatgtcccttgaaaatgtaagccagggtgtctctgtacactgacaagtcaacccaaataaaacgtgcacatgtaaaaccaaaaaaaaaaaaaaaaaa

***gadd45bb***

>gi|65301460|ref|NM_001012386| Danio rerio growth arrest and DNA-damage-inducible, beta b (gadd45bb), mRNA.

F primer: CAGCACTGAGAGAAAAATGG

R primer: TCATGAGTTGTGCAGACTCG

Fragment size: 113 bp

Annealing temperature: 50° C

Efficiency: 1.93

Source: [2]

aaaacacgagtcgagacagtgtcgcaccaagaggtatctacgagagagagaaaacccacacgaaacctctgtggattactactgccacacaacatcgacttattctgtggaattatacctactatttggtggaatttccagttgtggattataaacactttacggataaacatgactctggaggaagttgttggatgcaacagcactgagagaaaaatggagacagtgagtgaagctctggaggagctgctaatggctgcacagcgacaaaactgtctgacagttggagtctacgagtctgcacaactcatgaatgtggatccagacagcgtggtgctgtgcgttctagccaccgatgaggaggacgagaatgatgttgcgcttcagatccacttcacgctcatccaagccttctgctgcgacatcgacatcaacattgtgcgcgtgtccggcatgaggcgtctggcacaggttctcgaagagcctctgtccactgacaataacgccaatgaaccaagagaccttcactgcatacttgttactaacccccaagccgagcatctcaagctgaaggaagtgggaagttactgcaaggaaagccgctgcaggaaccagtgggtgccttctattgccctgcaagagcgctgaagtgtcgtgcactgaaatgtgctgaagagaaatggaatattgatgttgtccttcacctgtgaggacatgctcatcaattcaagaggactgtccaatagtcctttctggagcttctgcctccatgtttatggaattctaagtgtcagaggaaactagagacatttgtccacgttcctcataatgcatgatcggtgttatcgctcctctggacagaccaaaggagcatctgggtggtcggtggactgtgtcgtagctctgaaaggatgcagcaaggaatccaggggatatttccagacagcagcgtatggctacgctttgcactccacgtgggatgtgaatgaataacacatgactcagacctgacaatagtgtaggtctacagcggagaagctctgctttcaaaaagtgactgctatttaaagactgaatttttctttaatttatttatgcataacactgtcattttaaaaacgagtaatatatttggttttatttatgaatttgcacatgtttacctttcgttatgaaataaatgttatgcttaatttcaaaaaaaaaaaaaaaaaaaaaaaaaaaaaaaaaaaaaaaaaaaaaaaaaaaaaaaaaaaaaaaaaaa

***gapdh***

>gi|169403946|ref|NM_001115114| Danio rerio glyceraldehyde-3-phosphate dehydrogenase (gapdh), mRNA.

F primer: AAGTTGGTATTAACGGATTCGGT

R primer: GTAGACTCCACAACATAAGTAGCA

Fragment size: 283 bp

Annealing temperature: 53° C

Efficiency: 1.87

Source: [3]

actcacaccaagtgtcaggacgaacagaggcttctcacaaacgaggacacaaccaaatcaggcataatggttaaagttggtattaacggattcggtcgcattggccgtctggtgacccgtgctgctttcttgaccaagaaagtggagatcgtggccatcaatgacccattcattgaccttgattacatggtttacatgttccagtacgactccacccatggaaagtacaagggtgaggttaaggcagaaggcggcaaactggtcattgatggtcatgcaatcacagtctatagcgagagggacccagccaacattaagtggggtgatgcaggtgctacttatgttgtggagtctactggtgtcttcactactattgagaaggcttctgctcacattaagggtggtgcaaagagagtcatcatctctgccccaagtgcagatgcccccatgtttgtcatgggtgtcaaccatgagaaatatgacaactctctcacagttgtaagcaatgcctcctgcaccaccaactgcctggctcctttggcaaaggtcatcaatgataactttgtcatcgttgaaggtcttatgagcactgttcatgccatcacagcaacacagaagaccgttgatgggccctctgggaagctgtggagggatggccgtggtgccagtcagaacatcatcccagcctccactggggctgccaaggctgtaggcaaagtaattcctgagctcaatggcaagcttactggtatggccttccgtgtccccacccccaatgtctctgttgtggatctgacagtccgtcttgagaaacctgccaagtatgatgagatcaagaaagtcgtcaaggctgcagctgatgggcccatgaaaggaattctgggatacacggagcaccaggttgtgtccactgacttcaatggggattgccgttcatccatctttgacgctggtgctggtattgctctcaacgatcactttgtcaagctggtcacatggtatgacaatgagttcggttacagcaaccgtgtatgtgacctgatggcacacatggcctccaaggagtagatgtgacccctttgctgtttcttttttttgatacgcgaccattctcccatctggttgaatgtttgcaccacgtgcctggaaggaaattacatgcttaaattgaagaccaatattatttttatatactctgttctgtttcgtgtgtgaggttaaaaataaatgttgacttcaaaggcttttctgtctgttaacaacttgcgatggaataaaagtcctctgtttgtgagaaatgaaaaaaaaaaaaaaaaaaaaaaaaaaaaa

***gclc***

>gi|46358336|ref|NM_199277| Danio rerio glutamate-cysteine ligase, catalytic subunit (gclc),mRNA.

F primer: AACCGACACCCAAGATTCAGCACT

R primer: CCATCATCCTCTGGAAACACCTCC

Fragment size: 137 bp

Annealing temperature: 50° C

Efficiency: 1.95

Source: [4]

cgcgaggaagagccagacagaccgagctcgcgcactctgcgatcaaatatcagcctctcgccaccggagacccgctttatattcccccacataacgtgaaaccggagcccgtgctcgacctgagaggatccgtatttaaactcatcggtatttaaacccactcatataaggcctagcgtgagcgcgcgcgcgagcgagtttaacgttacattacgtcgggtttgttgttgcacgtgaagtgtgcgtaagttgacgtaaagaagagtacgctttttggggggaccgtaggttttgtgctatgggcttgctgtcacaggggtcaccgctcaactgggaagagaccaagaaacatgctgaccacgtaagaaaacacggcatcctgcagttcctcaacatctacaataaagtcaaagaccggcagaaggatgtgctcaagtggggagacgaggtggagtacatgttggtgaacatggatgaccaaaatgaaaaagtccgcctagttttgaatgggaaggaggttttggaaattcttcaggaaaagggtgaaaacatcaaccccaaccaccccacattgtggaggcctgagtatggcagctacatgatcgagggaacaccgggtcagccgtatggcgggacgatgtcggagttcaatacggttgaggataacatggggaagcggaggagagaggcttcatctgtgctcaagaagaacgaaactctcctcacagtcacggcattccccaggttaggctgcccgggcttcaccctgccggagtacaaacccacacctgttgaaaaaggggtttccaaatcgctgttcttcccagacgaggccatcaaccgacacccaagattcagcactctgaccagaaacatccgccataggaggggtgaaaaggtggtgataaacgtaccaatttttaaagatatcaacaccccgtctccattcatggaggtgtttccagaggatgatggagaagctgctcgaggtgccaagccggatcacatctacatggacgccatgggcttcggcatggggaactgctgcctgcaggtgaccttccaggcttgcagcatcagtgaggctcgatatctttacgaccagctggccactttctgtcccatagtgatggctctgagcgccgcctctccattctatcggggttttgtgtccgacatcgactgccgctggggtgttatctctgcatctgtggatgaccgtacccgagaggagagaggcctggagtcgctgaaaaacaacaaattcaggatccataaatcaagatatgattcgatcgacagctacctctcctgctgtggagagaaatacaatgacatcgagctgaccatagatgaggacatcaacaaacagctccttgatgcaggcattgacaaactgctggcgcagcacattgcacatctcttcatccgcgaccctctgtcgctcttccaggagaagattcacttggatgatgagaacgaatcggaccactttgagaacattcagtccaccaactggcagaccatgaggttcaagccccctccgccaaactctgagatcggatggagagtggagttcaggccaatggaggtccagctgacagatttcgagaactctgcttacgtggtgtttatcgttctgctcacccgcgtcatcctgtcctacaagctggatttcctcattccgttgtcgaaggtggatgaaaatatgaaggtagcccagaagcgaaatgccgtgcaggagggcatgttttatttccggaaggatgtctttaaaggctgtaccccagtgctggatcggccgggcgcagctcagaatggactggagactgaaactaatgatggagaagagtttatactgatgagcatcgacaccatcatcaatggaaaggaaggggtttttcacgggctgatccccatgttgaacagctatctggagaacatggaggtggacgtggacacacgctgcacaatcctcaactacctgaaactcatcaagaaacgagcttcaggtgatctgatgaccatggccaagtggatgagggagtttgttgccaaacatcctcaatataagcaggacagcgtcattacagacaaaatcaactacgacctgctccacaagtgtgacagaatcgcaaaaggagaagaaaaatgtccggaactgatcggagagccggtcaacagaggaaaatgaaggataatcatcgctaatcatatcaaatcatgcgaaagctcctctgcatgagagcgcaagaccaagtttgggacgcagcggtccagacaaagcaacgaaaatggaaacgttttaagagaatattgattttttttgaattgtgttgaatttgtaaatagcagattttttatgacgggcaaattattttaatagcttaaacagacgcaggatgttattgtacctgaacgtgtgtacatatttcacgccgctctgagcttgtgcgagattatgtaaatgtagcaaacactaaattgtacattagtcatattttcaatgccgaaatgtatatactgtacgccatcagacaataacaagtgtactttccatttcttttattttttatgaacgaaatttgcttatagttttggcatttattaatgaaccgagactgaagacagtgggttcggtgcagcactgactgatgttgaaactaatgcaagatgacagtagaagccttttaatggtgtgaagtgtgtcgtgtcactaacagatttactttcataactagtgtttttgataaatatgatattaggatatgattttccaccactagagggagcactggctctttttttatgacggaaatctatgaagatcgagcattgttatctgtatatggcttcagatgaagtgattcctgtacatattttcagcttacagagagatcttttctagagcgctgttgaaaatcatttgcattcaagccttgcgattgttttgaatgctgtttgccttgactgtttgtctgttcagtaaaactctgctcattaaatcatttaatcagcaaaaaaaaaaaaaaaaaaaaaaaaaaaaaaaaaaaaaaaaaaaaaaaaaaaaaaaaaaaaaaaaaaaaaaaaaaaaaaaaaaaaaaaaaaaaaaaaaaaaaaaaaaaaaaaaaaaaaaaaaaaaaaaaaaaaaaaaaaaaaaaaaaaaaaaaaaaaaaaaaaaaaaaa

***gpx1a***

>gi|169403975|ref|NM_001007281| Danio rerio glutathione peroxidase 1a (gpx1a), mRNA.

F primer: CAGATGAACGAGCTCCACAG

R primer: CCATTCACTTCCAGCTTCTCC

Fragment size: 185 bp

Annealing temperature: 53° C

Efficiency: 1.92

Source: this paper

tgcacttccttgacgcatttttacgcgcctcctattttcggcttcaaggttcaaatcaaggatcttgacggtaaataaaacctgcgtgttgccctttgagctgcagagaaaactgcgtgttcgagcatggcaggaaccatgaagaagttttacgacctgtccgcgaaactattgtcaggagatctcctgaatttttcctctctcaaaggaaaggttgtgctgattgaaaatgtggcgtcgctttgaggcacaacagtcagggattacacccagatgaacgagctccacagccggtacgccgaccaggggctggtggttctgggcgctccctgcaaccagttcgggcaccaggagaactgcaagaatgaagaaatcctgcagtctctgaaatacgtccgtcctggaaatggcttcgagcccaaattccaaattctggagaagctggaagtgaatggtgaaaacgcccaccctctgtttgcgttcctgaaggagaagcttcctcagcccagtgacgaccctgtgtcccttatgggagaccctaaatttatcatctggagccccgtgtgcaggaatgacatctcctggaactttgaaaagttcctcatcgggccggacggggaaccattcaagaggtacagcagaaggttcctcaccatcgacattgatgctgatattaaagagcttctgaagagaaccaagtaaaccagcggcttctacgtgttgtgttcttgcaagataaaccgtccactgcacatcacatgctttaaaaataagattgtagtttgatatagaccgactgtgcagtcatgattaaagtgccactgctttctacactgtttacttaatgaagatgcttcctaaaaccttttctgaggggagtttctgatggactgtaaatgtttattatagctgttgtaagttatgcatgaactgcacggctcaggttttatcattccactctgtgaactgaaaataaactttttaaacaaaaaaaaaaaaaaaaa

***gstp1***

>gi|124248588|ref|NM_131734| Danio rerio glutathione S-transferase pi 1 (gstp1), mRNA.

F primer: CCATTCACTTCCAGCTTCTCC

R primer: TGGCCAGAACATTTTCAAAGC

Fragment size: 428 bp

Annealing temperature: 50° C

Efficiency: 1.86

Source: [4]

NOTE: F Primer is located in 5' UTR and recognizes only gstp1. QGP probes are located in the coding region and recognize both gstp1 and gstp2.

agacgcgcgcgctccgacagtgcactgcagactaggagcaactttgaaacgcacttcactcagcgctacaacaccatggctccctacacactcacatacttcgcagtcaaaggcagatgtggtgctttgaagatcatgctggcggacaaagaccagcagctgaaggagaacctggtgacctttgaagagtggatgaagggcgacttgaaagccacctgtgtctttgggcagttgcctaaatttgaagatggtgacctggtgctgtttcagtccaacgccatgctgagacatctgggtcgaaaacatgctgcatatggcaaaaacgacagtgaggcttccctcattgacgtgatgaacgacggcgttgaagatcttcgcctgaagtacataaagctgatctaccaggaatatgagaccggtaaagaagcgttcatcaaagatctgcccaaccacctcaaatgctttgaaaatgttctggccaaaaacaaaaccggattcctggttggtgatcagatctcatttgcagactacaacctgttcgatctcctgctgaatctgaaggtgctttctccctcctgtctggactctttcccgtctctcaagagcttcgtggacaagatctctgcccgtcccaaagtcaaagctctgctggagtgcgagaacttcaagaaactgcccatcaacggcaacggcaaacagtaaatcaacactagacaacgtcggtacaacattctccaacaacgacacttactgctaaaacagccttaactggtgctcaatgatttgcttgatgtctcatgtctttgtatggaaatctattttgtatgtcttactgttttgatagtttttactaacagaaattccatgtatcagttattctgaataataaaaaaaaaaaccgtaaaaaaaaaaaa

***hmox1a***

>gi|189011581|ref|NM_001127516| Danio rerio heme oxygenase 1a (hmox1a), mRNA.

F primer: CCACGTCAGAGCTGAAAACA

R primer: AGCGCTCGGTAGATCTCGTA

Fragment size: 111 bp

Annealing temperature: 50° C

Efficiency: 1.94

Source: this paper

accctctctgctttgtcatgagaaagcgctgagatttcgcccagtcatgtttagctcaggcatgtgaaactggctcaacattttcacttccctttccactaaaaaaacagtgactcagacagagaggagaaagtataaaaacgaagtggggcggtcagggagctcacacagtcgcagtgaacactgaagacaacgcaagctctttaatatacagaaagaaagacaggacatctacagcacaaagatggactccaccaaaagcaaagcggcagagaacactggcagtgatctgtctgaacagataaaagcagtcactaaagacagccacgtcagagctgaaaacacacaactgatgctcagctaccagaaaggacagatcacacaaacacagtacaagcttctgctgtgctctctatacgagatctaccgagcgctggaggaagagctggacagaaacgcagaccacccagcagtgcagcccatttacttccctcaggaattggccagactggaggctctggggcaggacttggagcacttcttcggcccccagtggaggaagagaataacagtgcctgccgccacacaccgatatgcacagagactgagagagattggcaagagcagtcccgagcttctggtggcgcacgcttacacccgctacctcggagatctgtctggaggacaagttctgggcaagatcacacagaaatcactggggctgactggaaacaagggcattttattcttctcgtttcctggagtgacgagcgccaatagattcaagcagctgtacaggagcagaatgaacagcatagagttcacagagcaaaagaggcaggaagcactggacgaggccgtcagagcattcgagttcaacattgatgtgtttgatgatcttcagaaaatgctgagcatcacagaggaagcttcaagtgataaaggaaatgaagcagcatcccaaagtctgtcaaaaaccttctccagctctccagcccttcagttcgcattaggagtgggcatcacgttggcaacggttggcatgggagtttacgctttttaattcagattagttttttttactagtcatctgtaactgtaaatattaatacaatgaagtcaaatttacagactgatcattaaccaactgtaatgtatatgtactttatttctggatgcatttttcaacatgaaactgttaaaatacaaataatttttaaatgtatgtcggcttacaacttttgacagaatatgtcttcttaaaatgtattgttttaatacattaggcaaatgcttttccttaatgatttacagtgcatataccttcctgtccataaacgctttgagttgtcttctgtatgtattctgtgtaaatgcaagattaataaactgttttaatatatatttttaaaa

***hprt1***

>gi|47085696|ref|NM_212986| Danio rerio hypoxanthine phosphoribosyltransferase 1 (hprt1), mRNA. [Danio rerio]

F primer: ATGGACCGAACTGAACGTCT

R primer: GAGCGATCACTGTTGCGATT

Fragment size: 149 bp

Annealing temperature: 53° C

Efficiency: 1.93

Source: this paper

gaagagacaaattgtgcacgttcagtcctttaaaggtgaaattgagaattgtcagaagtacaactgcgtccggcatttcaccgagagttgaggaccgttttgcagtagcttgtcagagtggacggacatggcgtcatcgagcccgtgtgtcgtgatcagcgatgaggagcaaggttatgacctggacctcttctgtataccaaaacactatgcagctgacttagagcgggtgtatattcctcacgggctcatcatggaccgaactgaacgtctggccagagatatcatgaaggacatgggtggacaccatatagtggctctatgtgtgctcaaaggaggctacaagttttttgctgacttactagattacatcaaagcccttaatcgcaacagtgatcgctccattcccatgacagtggacttcatccgcctcaagagttaccaaaatgaccagtccacaggtgacatcaaagtgattggtggagatgatctgtccacgctaacaggaaagaacgtcttgattgtggaggacatcattgatactgggaaaacgatgaaaacgctgctagaacttctaaagcaatataacccaaaaatggtcaaagttgcaagtttgctggtgaagaggacaccgaggagcgttggttacagaccagactttgtaggatttgaagttcctgacaaattcgtggttggatatgcacttgactacaacgagtactttagagatttaaaccacatctgtgtgatcagcgaaacaggaaaggagaagtacaaagcatgagaagccatgaacttccccctctagctgtgtatcatttgtttttaatagacgtcttacatttttacatccattttatatatatatatatattttgttttgtttttcagtagtgcagctcaccgcaggcacccaacaaacatcatacatcaaccacaaacactttgcagagtttagcctctgagaacagaggtctacaagctgctgctcttggtggaaagaagcgtttaaacacctagttgtgatagggccagaatccctttaatttattataatgatctgcacgagatgttataggtaggtgtttgttttaaagattaaagtgaaaattgactttaggtcatcttgcttgctattcaatagtgcacatagaattggtttgctcaaatggctgaagtagaacattcacctttccaaagactctttgcagtgcataactgtaataggaacaatgttattaatgatctagatctgtgggtaatgtgattacatacaatatgttgaatgcttttacatgtaaatgtacttttagcgtcaggacaacctgatcatccaatcacatttcaaatttaaagccaaatgttttttagggttcctgcaggttttatgaaagtaaatttaagacttttttaaagagttttactttgtccaagtaaagaaattatgattaattttaagagaatgcaatatgtaaatgtttattgtaaatttaaagggagcacacattgagatgtattagtttaaaaatgcatatccttttatttaatttaaattaatttgacatcacatgtttggtttcaaccacttgactaggcaaatatttttgtacctctgatcacactgcaaaagtcatgttttatcaatatatttgtcttgttttccagtgcaaatgtctacaaattattaaatcaagccacatttacttaaaaaaaaaaaaaaaaaa

***hsp70***

>gi|828747278|ref|NM_131397| Danio rerio heat shock cognate 70-kd protein, tandem duplicate 3 (hsp70.3), mRNA.

F primer: GCACAAGAAGGACATCAGTCAGA

R primer: GGATGCCCTCGTACAGAGAGT

Fragment size: 131 bp

Annealing temperature: 50° C

Efficiency: 1.95

Source: [5]

NOTE: qPCR primers have 100% sequence homology to all three isoforms of hsp70. QGP oligos have 0-1 nt difference in sequence to each of the three isoforms (detailed below). All QGP oligos likely bind to all three isoforms.

ttcgcgatagaactgtatacagcggaaagcgagacagcgagcggactgggccatccagacaacaagccatcaatacgcctgacataaaagatccaacaaacataaaagtttcttgaaatcaatcatgtcctctccaaaaggaatcgctattggcattgacctgggcaccacctactcctgtgtgggggtgtttcagcatggaaaagtggagatcatcgccaacgaccagggcaacagaacaacacccagctatgttgccttcacagacacagagaggctgattggagacgcagctaagaaccaggtggccatgaatcccaacaacacggtgttcgatgccaagaggctgatcggcaggaggttcgatgaccctgtagtgcagtctgacatgaagcactggtccttcaaagtcgtcagtgatggaggaaagccaaaagttgcagtggaacacaaaggagaaaacaagacctttaatcctgaagagatttcctccatggtcctggtgaagatgaaggagattgcagaggcttatctggggcagaaggtgaccaacgcagttatcacagttccagcctatttcaacgactcccagagacaggccactaaagatgctggagtcatcgctggactgaacgtgctccgcatcatcaatgagcccacggctgcagccatcgcctacggcctggacaaaggcaaatcctcagagcgcaacgtgctgatctttgacctgggcggaggcaccttcgacgtgtctatcctgaccatcgaagacggcatctttgaggtgaaggccaccgctggagacactcatctgggcggtgaggactttgacaaccgcatggtgaaccactttgtggaagagttcaagaggaagcacaagaaggacatcagtcagaacaagagggccctgaggagactgcgaaccgcatgcgaacgagccaagaggacgctctcgtccagctctcaggccagcattgagatcgactctctgtacgagggcatcgacttctacacgtccatcaccagagctcgcttcgaggagctctgctccgacctcttcaggggaacgcttgatcctgtggagaaagcactgagggacgccaagatggacaaggctcagatccacgacatcgtgctggttgggggatcaacaagaatcccaaagatccagaagcttctgcaggatttcttcaacggcagagaactgaacaagagcattaaccctgatgaagcggtggcttacggtgccgcggtgcaggccgccatcctcatgggcgacacctctggaaacgtgcaggacctgctgctgctggacgtggccccactgtctctgggtattgagaccgcaggtggagtcatgacggccctcatcaagcgcaacacaactatccccaccaaacagacccagaccttcaccacctactccgacaaccagcccggcgtcctgatccaggtgttcgagggagaaagagccatgaccaaagacaacaacctgctgggcaaatttgagctgacgggaattccacctgcgccacgtggcgtcccgcagatcgaagtgaccttcgacatcgacgccaacgggatcctaaatgtgtcggcggcggacaaaagcaccggaaaacagaacaagatcaccatcaccaacgacaagggcaggctgagcaaagaggagatcgagagaatggtgcaggaggccgacaagtacaaagctgaagacgatctgcagagagagaagatttctgccaaaaactccctggagtcttacgccttcaacatgaagaacagtgtggaagacgacaacctgaaaggcaagatcagcgaggaggacaagaagagggttattgagaagtgcaatgaagccgtgagctggctggagaacaaccagctggcggataaagaggagtacgaacatcagctgaaggagctggagaaagtctgcaatccagtcatctccaaactctaccagggagggatgccagctggaggatgtggagctcaggcacggggcgcatcaggggccagcgctcaggggcccaccattgaagaggtggattaaaactcctcatgaactgaacaaactagacaaaaaaataattctttgattattttaagatgactttatttaaagtcttattgcacagtgtgttggttcactatctacttaaacatcttgatacagtaaaatgtttacatttattaaataatgttaaactttaatttattttttgtttagaccaaaaataagcttgaccattttaagaaattgtagtttagttttcatttttcattcatgttcatgttgttacatgtgacaaatttgtgataaaaatgtgttaataaaagtgtttgtttttttaaaaaaaaaaaaaaaaaaa

LE tggaggaaagccaaaagttgc found in all 3

CE agtggaacacaaaggagaaaacaagacctttaatcctgaagagatttcctccatggtcctggtgaagatgaaggagattgcagaggcttatct found in all 3

LE ggggcagaaggtgaccaac found in hsp70.1, hsp70.3

CE gcagttatcacagttccagcctatttcaacgactcccagagacag found in hsp70.1, hsp70.3

LE gccactaaagatgctggagtca found in all 3

CE tcgctggactgaacgtgctc found in all 3

CE aaatcctcagagcgcaacgt found in all 3

LE gctgatctttgacctgggcg found in hsp70.2, hsp70.3

LE CGCATCATCAACGAGCCC not found in the sequence, possibly due to NCBI sequence changes (found in hsp70.1, hsp70.2) (CGCATCATCAA**T**GAGCCC found in hsp70.3)

CE ACGGCTGCAGCCCATCC not found in the sequence, possibly due to NCBI sequence changes (not found in any) (ACGGCTGCAGCC-ATC**G** found in all 3)

LE GTACGGCCTGGACAAAGGC not found in the sequence, possibly due to NCBI sequence changes (not found in any) (**C**TACGGCCTGGACAAAGGC found in all 3)

CE GAGGCACCTTCGACGTGTCC not found in the sequence, possibly due to NCBI sequence changes (found in hsp70.1) (GAGGCACCTTCGACGTGTC**T** found in hsp70.2, hsp70.3

***nqo1***

>gi|324021696|ref|NM_205542| Danio rerio NAD(P)H dehydrogenase, quinone 1 (nqo1), transcript variant 2, mRNA.

F primer: CGAGATGTTGCAGTTCAGGC

R primer: ATCGACCCTCTTTCCATGCA

Fragment size: 175 bp

Annealing temperature: 53° C

Efficiency: 1.90

Source: this paper

gagaccggtcacacgagattgtccctgcgggtgtgcagaaaaagagtacacagtagtacagaatacagatgtggtagcagagagtttgccgattcaccattcgactgcaaagtgtcaaagttacacaataacagatactgaaacctgagacagaatcagacaccacttcacacgcgaggggtttgcactggtataaaggcagttcattgagaagcgttcactcatctatcctggtgcagaatttgagattgcaaacaaaaagaaatggcacagaagaccgcactgatagtttatgctcatcagagtcctgcctccttcaacgccgctgcgcgagatgttgcagttcaggctctgacaaagaaaggctacaaagtccttgtttcagatctctatgctatgaaattcaaggcttcagctactgcggaggatattaagggtgatttgcagaatcccgagcactttgtgtataataatgagatgatggttgcatggaaagagggtcgattaagtgacgatgttgcagaagagcagcacaaggtggagcaggcggatctaattatctttcagtacccactctactggtttaccatccctgccatcatgaagggctggatcgaccgagtcttaactcaaggatttgccttcagcatgcagaacatgtatgacaacggcattttcaagaataagagggccatgctttccttcaccactggaggaatggagtccatgtataaggatgatagccttcatggagacatcaacattctcctctggcccttacagaatggagtgctgcgtttctgcgggttccaggtcctcgcccctcagatcttctggtctcctgcatacacgcctccagagggaagagctgccatgctggatggatggcgagagaggctgggtggtgtgtttgaagaaaagcctctgtcctttgctccgtctgaatattttgaccttagctttcaagcaggattttgtctccggcctgaagttaaagataagttggcttctgaaccctacggcattaccacagcacatcatttaggaaaacctctcccaccaaacaaccagaccaaacctaagcaaaactaaaggatccagcgtagctaccaatacatcactcattactacgtatagcagatttataaagctttaggtgaaatgtgctccagatcaaaaatattctattctgtcaataatctaaactatactgtctacctgtcatttatgctttataactaaaattatctaccagcaaataagccatacgactgttgggacaatcagctaaatgtaatttttctgatgtctcttgttattttcgtagatagttttactaatatcggtgtcttgatgaagtaacttgtcaaataaagattctaatgctgttgtgatttgaatgttgtttaatatatcatatattataatgatcatggcgatgcttgtggtgcttttttagattctggttatgcaaatacaaataaataactataactgaa

***prdx1***

>gi|606215143|ref|NM_001013471| Danio rerio peroxiredoxin 1 (prdx1), mRNA.

F primer: TTTCATTCATTCACTCTCGGATT

R primer: AAACTGTCCATCAGGCATCAC

Fragment size: 120 bp

Annealing temperature: 60° C

Efficiency: 1.93

Source: [4]

aaagtcatgctgccataaacacttctctgtaatttggtctttccacggagcatttactcctcggagcagcgcatcttgcagagtatcgcgagacttgagcacgacctatttaaacaccagccgctgcccgcgagttcactttcattcattcactctcggattgtgaagacagttcaagaaaatggcagctggaaatgcacacattgggaaacctgctccggatttcacagccaaagcagtgatgcctgatggacagtttggagatgtccgtttgtctgactacaaagggaagtatgtggtgctgttcttctatccactggatttcacctttgtctgtcccactgagatcatcgccttcagtgatgctgccgaggagttcaggaaaatcaactgcgagatcattggtgcttctgtcgattcccacttctgccatcttgcctggaccaaaacaccccgaaaacaaggtggtttaggaccaatgaatgtccctctggtggcagatactctccgctccatttctaaagactacggtgtacttaaagaagatgagggtattgcatacaggggtcttttcattattgacgacaagggcattctgcggcagattaccatcaacgatctgccagtcggtcgctccattgatgaaaccctgcgcttggtgcaggcctttcagttcaccgataagcatggagaagtttgtccagccggatggaagcctggaaaagacactattaagcccgatgtcaatcagagcaaagacttcttctccaagcaaaattaaaactaaaaaaggcactagactctgcagcatagtataatagacagaagtgatgtgaattaatgctactggttggactagtacatccatttacaggtggttattcttattatatagtgttatatattgtaaagttgtaggactaaagtgtgtcagactacctgaaatgtattgaaccgtttgagttggtttgttcaactttgtcttaccatttagttttgtgtgaaaagagatgttttatagcgatgaccttctgactacactgtcaacaattgcatttcaaataataaactcgtattttgtaaaagctttattagtttatttgttaccactgtttgatttgtaaacatttataaatcttcagtgctacatgtttttcttggtctgttgttatcggatgtgttaagagtgtcgtaaagtaacaaattacaaacactctgtactactaaaaaaaaaaaaaaaaaaaaaaaaaaa

***sod1***

>gi|56790261|ref|NM_131294| Danio rerio superoxide dismutase 1, soluble (sod1), mRNA.

F primer: CGCACTTCAACCCTCATGAC

R primer: TGAATCACCATGGTCCTCCC

Fragment size: 137 bp

Annealing temperature: 50° C

Efficiency: 1.90

Source: this paper

tcttatcaaacacagtcggtttctttcactctctcacaacttctcagtttgcataatctacagtcagcatggtgaacaaggccgtttgtgtgcttaaaggcaccggtgaagtgaccggcaccgtctatttcaatcaagagggtgaaaagaagccagtgaaggtgactggtgaaattactggccttactccaggaaaacatggtttccacgtccatgcttttggtgacaacacaaacggctgcatcagtgcaggtccgcacttcaaccctcatgacaaaactcatggtgggccaaccgatagtgtgagacacgtcggagacctgggtaatgtgaccgctgatgccagtggtgttgcaaaaattgaaatcgaggatgcaatgctaactttgtcaggccaacattctattattgggaggaccatggtgattcatgagaaggaggatgacttggggaagggtggcaatgaggaaagtcttaaaactggcaacgctggcggtcgtctggcctgtggagtgatcggcatcactcagtgaatctgctctaatggaagagccggttgaaatattggtgaccaatgtggatgcctctgaaagcacttagcccgctgacaattacatctctatttttgttaggtaacgttgaaagaattgtactttagccttgttaagctgtttttctgtgcatttctatgtgggcaaccttttatttgttatctgcttaattcattaaacattgagcagactggaaaaaaaaaaaaaaaa

***sod2***

>gi|41152469|ref|NM_199976| Danio rerio superoxide dismutase 2, mitochondrial (sod2), mRNA.

F primer: ACTGTGTGACGGACTAGAGC

R primer: CAGATGTGAGGCTCAAGTGC

Fragment size: 154 bp

Annealing temperature: 50° C

Efficiency: 1.94

Source: this paper

cttaggtctgttggttggtcgcttgtatcactgtgtgacggactagagcatgctgtgcagagtcggatatgttcggaggtgcgctgcaaccttcaaccccctgttaggtgctgtgacctccagacagaagcacgctctccctgacctcacatatgactatggtgcacttgagcctcacatctgtgctgagattatgcagcttcatcacagcaagcaccatgcaacatatgtcaacaacctcaatgtcacagaggagaaatatcaagaggctctggccaagggtgatgtgacaacccaagtctcccttcagcctgcattgaaatttaatggaggtggtcatattaatcataccatattctggacaaatctgtcacccaatggcggtggagaaccacagggtgagctgttggaggccataaagcgtgactttggctcatttcagaagatgaaagagaagatatcagctgccaccgtggctgttcagggctcaggctggggctggctgggctttgaaaaggagagcggaagattgaggattgcagcgtgtgctaaccaagaccctttgcaagggaccacaggtctcatcccactgcttgggatagatgtctgggaacatgcgtactatctccagtacaagaatgttagaccggactatgttaaagccatctggaatgttgtaaactgggagaatgtcagcgagcgtttccaagctgccaagaaataaataaattgcaccacacttgggagagaaaagagagcaaccgagtgtctattttgtactttcgaagcgcacaattaatcagaccacgatacagctatcaaacaaaactgaggcttggtattgttcttgacaagctataatccatacagaactgtgcactgtaatgcgtagtttgcattgagttattgatgtacgataatgctttcagtgtattcaaattaaaaggttatttgaacagtcaaaaaaaaaaaaaaaaaa

1. Timme-Laragy AR, Van Tiem LA, Linney EA, Di Giulio RT. Antioxidant responses and NRF2 in synergistic developmental toxicity of PAHs in zebrafish. Toxicological Sciences. 2009;109: 217–227. doi:10.1093/toxsci/kfp038

2. Hahn ME, McArthur AG, Karchner SI, Franks DG, Jenny MJ, Timme-Laragy AR, et al. The transcriptional response to oxidative stress during vertebrate development: effects of tert-butylhydroquinone and 2,3,7,8-tetrachlorodibenzo-p-dioxin. Tanguay RL, editor. PLoS ONE. 2014;9: e113158. doi:10.1371/journal.pone.0113158.s005

3. Sarkar S, Mukherjee S, Chattopadhyay A, Bhattacharya S. Low dose of arsenic trioxide triggers oxidative stress in zebrafish brain: expression of antioxidant genes. Ecotoxicology and Environmental Safety. Elsevier; 2014;107: 1–8. doi:10.1016/j.ecoenv.2014.05.012

4. Wang L, Gallagher EP. Role of Nrf2 antioxidant defense in mitigating cadmium-induced oxidative stress in the olfactory system of zebrafish. Toxicology and Applied Pharmacology. Elsevier Inc; 2013;266: 177–186. doi:10.1016/j.taap.2012.11.010

5. Kreiling JA, Creton R, Reinisch C. Early embryonic exposure to polychlorinated biphenyls disrupts heat-shock protein 70 cognate expression in zebrafish. Journal of Toxicology and Environmental Health, Part A. 2007;70: 1005–1013. doi:10.1080/15287390601171868
